# Feature-reconstructed diabetic retinopathy classification using variational autoencoder with disentanglement factor

**DOI:** 10.3389/fmedt.2026.1826143

**Published:** 2026-07-20

**Authors:** Ashu Priya, Niranjana Banerjee, Manas Ranjan Prusty

**Affiliations:** 1 School of Computer Science and Engineering, Vellore Institute of Technology, Chennai, India; 2 Centre for Cyber Physical Systems, Vellore Institute of Technology, Chennai, India

**Keywords:** diabetic retinopathy, disentanglement factor, features focused mechanism, logistic regression classifier, severity grading, variational autoencoder

## Abstract

Diabetic Retinopathy or DR is one of the leading causes of blindness in the working age population across the world, making its early detection and accurate classification as one of the major challenges in healthcare and medical imaging. Over the years various approaches have been developed like conventional deep learning models to classify DR or grade DR according to its severity. However, it has been observed that some of the major challenges encountered by various approaches is the lack of a lightweight feature focused learning mechanism. In this study, the authors propose a Variational Autoencoder (VAE) with disentanglement factor (Beta) and Logistic Regression based framework to classify DR in both ways, binary and multilevel classification. The proposed architecture aims to extract the fine features and textures of the retinal images compress it into a latent vector via the encoder and then expand the image map into a reconstructed image which aids the Logistic Regression classifier to classify the images and distinguish the severity of the disease. The proposed model was evaluated on two well-known datasets, APTOS 2019 dataset and DDR dataset. In case of binary classification, the model achieved an accuracy of 98.64% on the APTOS 2019 dataset and a 97.83% accuracy when evaluated on the DDR dataset. On multilevel classification, the model recorded an accuracy of 97.80% and 97.23% on APTOS 2019 dataset and DDR dataset respectively. These findings highlight the potential of the proposed method as an accurate and effective tool for automated DR screening and severity grading.

## 1 Introduction

Diabetic Retinopathy (DR) is a microvascular complication which is often a result of diabetes mellitus (1). Diabetes adversely causes damage to the blood vessels in the body due to elevated sugar levels in the blood, which leads to changes in the tiny blood vessels located in the retina. DR, if left undiagnosed and untreated for a long duration, can lead to other complications such as Diabetic Macular Edema (DME) or Neovascular Glaucoma. DME occurs when blood vessels in the retina start leaking fluid, which causes blurry vision. Neovascular Glaucoma is a condition that results from blood vessels growing out of the retina and blocking the fluid from draining out of the eye. It is a leading cause of vision loss and blindness, especially in the elderly, with one million people being diagnosed as blind due to DR. DR can advance from Mild Non-Proliferative DR to Proliferative DR, which can then lead to DME (1). Moderate NPDR is often characterized by leakage of retinal blood vessels and intraretinal hemorrhage, which can be easily prevented by early detection.

**Figure 1:**
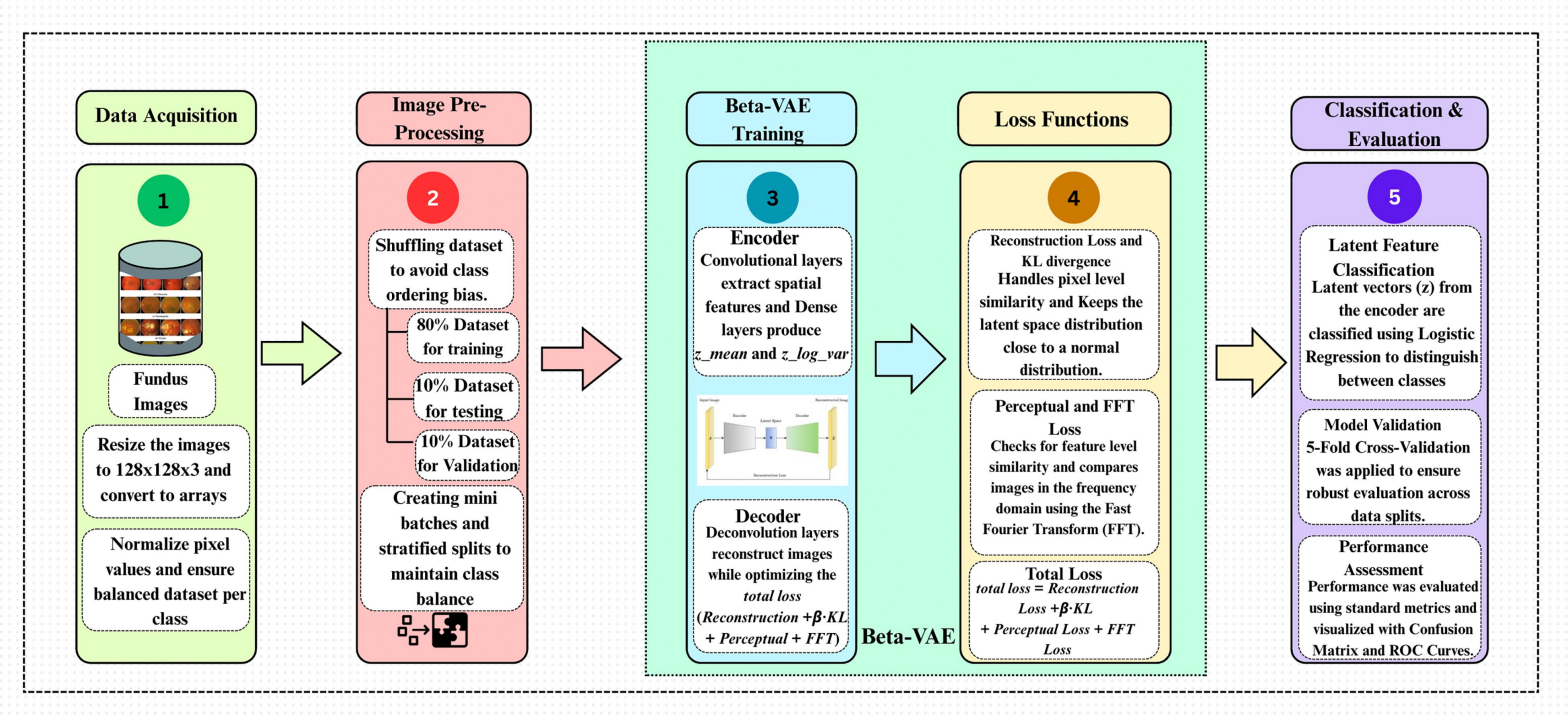
Workflow of proposed approach.

Apart from the serious complications of DR, the growing prevalence of DR in certain regions of the world should also be noted. Lin et al. (2) state that in Taiwan there has been an increase in diabetic eye diseases from 3.75% to 3.95%, and growth has been recorded in poor vision and blindness from 0.295% to 0.35% from 2005 to 2014. Early detection of the disease, along with timely intervention and identification, is a key factor in slowing down the advanced stages or preventing them (3). Limitations in medical infrastructure, proper resources, and screening remain crucial factors that slow down the diagnosis and treatment of DR. Traditional methods of diagnosis, such as a dilated eye examination, can be time-consuming, and proper training, experience, and expertise are required for accurate diagnosis. In recent years, Artificial Intelligence (AI) and Machine Learning (ML) technologies have contributed extensively to the early detection of DR by providing accurate and reliable methodologies to identify and detect DR at various levels using diverse techniques. Medical imaging and Computer Vision techniques have greatly improved by leveraging efficient methods to detect DR, such as using Optical Coherence Tomography (OCT) on fundus images (4). Zhou et al. (5) developed a framework that segmented the retina from the background of OCT images using a U-Net network, and then Spatial VAE (S-VAE) and Contextual VAE (C-VAE) were used to reconstruct the retinal images and detect crucial anomalies. Chokuwa et al. (6) proposed a method of utilizing a VAE with domain-invariant representations to tackle domain shift in datasets.Various models such as VGG16, DenseNet-169, CNN512, and YOLOv3 have achieved high efficiencies by training and testing on retinal fundus image datasets. However, a lightweight and simpler approach can be crucial in reducing computational cost, time complexity, and memory requirements while ensuring accuracy and reliability. This paper proposes a two-stage framework for DR classification using reconstructed images from a Beta Variational Autoencoder (Beta VAE), utilizing reconstructed images that are denoised, simplified, and contain only the essential features that help in the identification of DR. In Figure 1 given below, the approach is broadly divided into Data Acquisition, Image Preprocessing, Beta VAE Training, Loss Functions, Classification, and Evaluation. The reconstructed image retains the essential features that are instrumental in the detection of DR.

The rest of the paper is organized as follows. The Literature Review in presented in section 2 and the Motivation and Objectives are presented in section 3. The Proposed Methodology is elucidated in section 4 followed by the Results and Discussion in section 5. Finally, section 6 shows the conclusion.

## 2 Literature review

In recent years, Convolutional Neural Networks (CNNs) (7) have been widely adopted for most automated DR systems. Long et al. (8) have used a self-supervised learning approach that used a VGG-16 network with nine branches to learn the integral features of fundus images, achieving 92.7% accuracy on the binary APTOS dataset. Similarly, machine learning-based methods, such as employing a pre-trained model Inception V3 (9), achieved an accuracy of 81.61% on binary datasets. DenseNet architectures, which include DenseNet-169 (10) and DenseNet-201 (11), are densely connected convolutional networks that classify fundus images based on severity levels: No DR, Mild, Moderate, Severe, and Proliferative DR. To overcome the limitations of cross-entropy loss, Islam et al. (12) have proposed Supervised Contrastive Learning (SCL) as a two-stage training method that learns more distinct features. Recently, efficient models such as EfficientNet-B7 (13) have been used for automated DR detection, showing that preprocessing and augmentation techniques such as Usuyama preprocessing and hyperparameter tuning can significantly enhance model performance and reduce overfitting. A decision tree-based ensemble learning technique (14) extracts gray-level intensity and texture features from retinal fundus images to classify DR. In addition, clustering and optimization algorithms, including Fuzzy C-Means (FCM), k-means, Deep Embedded Clustering (DEC) (15), and Differential Variational Embedding (DVE), have been included to refine feature spaces and enhance the separation of DR severity levels.

More recently, Burr and Iskander et al. (16), addressed the challenges of high-resolution fundus image analysis under GPU memory constraints by proposing MEDCNet, a memory-efficient divide-and-conquer CNN-based method. Source-Free Transfer Learning (SFTL) (17) has been introduced to address the challenges of medical annotation and privacy issues, as it eliminates the need for labeled target data. Tudela and Hornero et al. (18), in their paper, used a modified ResNet-50 architecture, and in order to make the model explainable, SHapley Additive exPlanations (SHAP)-based visualizations were applied, generating heatmaps that highlight vascular changes and peripheral retinal regions as key indicators of DR progression.

Overall, existing studies on DR detection span a wide range of strategies, from CNN baselines to hybrid ensembles, clustering methods, transfer learning, and interpretability frameworks. Although these approaches have yielded good results, many depend heavily on large annotated datasets or involve complex architectures that increase computational cost. These challenges emphasize the demand for models that can learn from limited annotations, generalize across datasets, and maintain transparency. A Variational Autoencoder (VAE)-based model is therefore proposed to improve feature embedding and early detection of DR.

## 3 Motivation & objectives

The following section establishes the motivation and objective for this paper. We begin by discussing the motivation to address the issue of proper classification by pattern analysis and then detail the objectives of our research.

### 3.1 Motivation

Traditional deep learning models usually incorporate methods to compress images but lack mechanisms to capture and analyze the instrumental latent features that play a key role in image classification, object detection, and image retrieval. Variational Autoencoders (VAEs) aim to identify patterns in images that help in the efficient classification of DR. The major issue with prevalent encoder designs is that they either employ mechanisms with a large number of parameters or fail to capture the intricacies of the data. This highlights the requirement for an encoder that not only works as an efficient compressor but also utilizes techniques that ensure the extraction of features aiding classification while being computationally fast and memory optimized.

Apart from the mentioned issues, the latest research reveals that deep learning algorithms struggle with detecting fine distinctions between classes (19). Besides, they usually ignore local lesions and other minor features such as microaneurysms and bleeding, which are crucial for an early diagnosis yet fail to be detected through traditional convolution processes (20). Another problem is the necessity of applying a large number of preprocessing methods in order to highlight the features. This means that deep learning models cannot recognize relevant patterns in raw images without additional manipulation (21). Most approaches in the field use a single modality, such as fundus images. But this ignores other sources of information, such as medical history or multimodal data (22). Finally, deep learning-based systems demonstrate inconsistent results depending on the imaging equipment and lighting used during image acquisition (23).

### 3.2 Objective

The objective of this research is to develop a lightweight encoder design that extracts important features from the image data. The key aspects are as follows.

• The proposed encoder includes two convolutional layers that carefully analyses the patterns in the image.

• A down sampling of the image is applied to ensure that its key features are still intact.

• These key features are essential in the classification of DR into its levels of 0,1,2,3,4 which span from No DR to Severe.

• This down sampling is integral for maintaining the lightweight approach while absorbing useful features.

• Separate dense layers for mean and variance are kept facilitating independent and accurate learning.

### 3.3 Novelty and contributions

This work proposes an efficient and clinically relevant framework for diabetic retinopathy (DR) classification by focusing more on improving the feature representations feature representations instead of increasing the model complexity. The novelties over the existing approaches proposed in the paper are as follows:

- Feature reconstructed learning mechanism: The proposed approach introduces a reconstruction-based learning in which the classification is done on the reconstructed images instead of the raw fundus images. These reconstructed outputs keep the disease specific patterns intact such as lesions and vascular variations white suppressing noise and irrelevant background information. This makes the model to focus on clinically significant features which in result improve the performance
- Disentangled Latent Representation using beta VAE: In the proposed approach a beta Variational Autoencoder has been used to learn disentangled latent representations of retinal images. This helps in effectively separating the meaningful features related to the severity of the disease from the irrelevant variations which in turn helps to improve the model's ability to distinguish between the different stages of diabetic retinopathy.
- Lightweight two stage pipeline: The proposed method uses Beta VAE along with a Logistic Regression classifier which creates a simple two-stage pipeline that performs good without being too complex. It achieves high accuracy while keeping the model simple and faster to run. This approach requires less computation and is more practical for real-world use compared to the larger models like EfficientNet, DenseNet, or ensemble CNNs,
- Dual dataset evaluation: The model is tested on two different datasets, APTOS 2019 and DDR, for both binary and multi class classification using 5-fold cross-validation. This helps in ensuring that the results are consistent and not limited only to a single dataset. This also ensures that the model can handle differences in image quality, data collection conditions, and patient variations. The consistent performance across both the dataset indicates that the model is stable and can manage class imbalance. It can closely relate the classes effectively. It also reduces the chance of bias making the approach more reliable for daily use.

## 4 Proposed methodology

The suggested method's overall workflow as per Figure 1 starts with the collection of retinal fundus images, which are then prepared by resizing, normalizing pixel values, and making sure that class distributions are balanced. Following acquisition, the images are pre-processed by shuffling the dataset to eliminate ordering bias, dividing it into training and testing sets, and then creating stratified mini-batches to preserve equal class proportions. Following processing, these images are fed into the Beta-VAE model for training. The encoder uses convolutional layers to extract spatial features and map them into latent variables (mean and log-variance), and the decoder uses this compressed representation to reconstruct the images. Reconstruction loss, KL divergence, perceptual loss, and FFT loss are among the losses that are optimized during the training process to guarantee that the reconstructed images preserve crucial retinal features like lesions and textures. A Logistic Regression classifier that performs both binary and multiclass DR classification receives the flattened reconstructed outputs as input. The model's robustness and generalization across data splits are then assessed using 5-fold cross-validation and metrics like accuracy, precision, recall, and ROC-AUC.

To test the significance of the study's results an Anova test was performed. The Anova test helps to determine which of the hypothesis is true, namely null and alternative hypotheses. While null hypothesis ($H_{0}$) states that there is no modification in the means of the significant group, alternate hypothesis ($H_{A}$) states there is a noteworthy difference between means of the significant groups. For our study the one-way Anova is specifically considered. The formula below represents the test statistics:

Sums of squares are measures of variation in the context of the Anova test. The sum of squares within is defined as the sum of variations that are observed within each of the several groups. This value is computed as shown above in Table 1, where $X_{j}$ represents the individual values present in the group and $X_{j}^{\prime }$ represents the group mean of the element belonging to that group. SBB, referring to the sum of squares between i.e, the variations observed between the group, is formalized by computing the difference between the group mean $\left(X_{j}^{\prime }\right)$ and the grand mean $\left(X^{\prime }\right)$ for each subject. The sum of squares of total (SST) is represented as the difference between each score ($X_{j}$) and the computes grand mean ($X^{\prime }$). SST can also be defined as the sum of SSW and SSB. The degrees of freedom for the different sources of variation is shown in Table 1. Here k represents the number of samples involved with one data value for each group i.e., the sample mean and *n* denotes the total sample size.

**Table 1:** Anova test statistics result formulation.

Source of variation	Sum of squares	Degrees of freedom	Mean squares	F-value
Within	$SSW=\overset{k}{\underset{\;j=1}{\sum }}\;\overset{l}{\underset{\;j=1}{\sum }}\;(X_{j}-X_{j}^{\prime }{)}^{2}$	$df_{w}=k-1$	$MSW=\frac{SSW}{df_{w}}$	$F=\frac{MSB}{MSW}$
Between	$SSB=\overset{k}{\underset{\;j=1}{\sum }}\;(X_{j}^{\prime }-X^{\prime }{)}^{2}$	$df_{b}=n-k$	$MSW=\frac{SSW}{df_{b}}$	
Total	$SST=\overset{n}{\underset{\;j=1}{\sum }}\;(X_{j}-X^{\prime }{)}^{2}$	$df_{t}=n-1$		

### 4.1 Data acquisition

To examine the performance of the proposed model, we have used retinal fundus image datasets that contain images with different levels of diabetic retinopathy severity. These datasets have been widely used in the literature, making them baselines for comparing new methods with existing state-of-the-art approaches. In this study, we have used two datasets: APTOS 2019 Blindness Detection and the DDR dataset.

APTOS 2019 Blindness Detection (24): The APTOS 2019 dataset was released as part of the Kaggle Blindness Detection Challenge organized by the Asia Pacific Tele-Ophthalmology Society (APTOS). It contains 3,662 retinal fundus images that have been annotated into five severity levels: No DR, Mild, Moderate, Severe, and Proliferative DR. Each image has a severity label for diabetic retinopathy, which is provided in the train.csv file.

DDR Dataset (25): The DDR (Diabetic Retinopathy Dataset) contains 13,673 fundus images collected from 147 hospitals across 23 provinces in China, providing diversity in patient demographics and imaging conditions. Each image is categorized into five levels of severity: No DR, Mild, Moderate, Severe, and Proliferative DR, along with CSV annotation files that contain the labels, with poor-quality images excluded from the released version to ensure reliability. With its size, quality, and comprehensive labelling, the DDR dataset has become a widely used benchmark for developing and evaluating automated systems for diabetic retinopathy screening.

The images from both datasets are organized according to the CSV files provided with the datasets into folders corresponding to the five severity levels. However, both datasets suffer from significant class imbalance, with No DR being much more frequent than higher severity categories. Therefore, class balancing techniques are applied by undersampling the majority class and augmenting the minority classes through rotations, flips, shifts, and zooming to improve representation. Alongside the original multi-class classification setup, a binary classification setting is also created by merging all the diseased categories (Mild, Moderate, Severe, and Proliferative DR) into a single DR class, while retaining No DR as the second class, thereby allowing the model to be evaluated for both binary detection and detailed severity grading.

**Figure 2:**
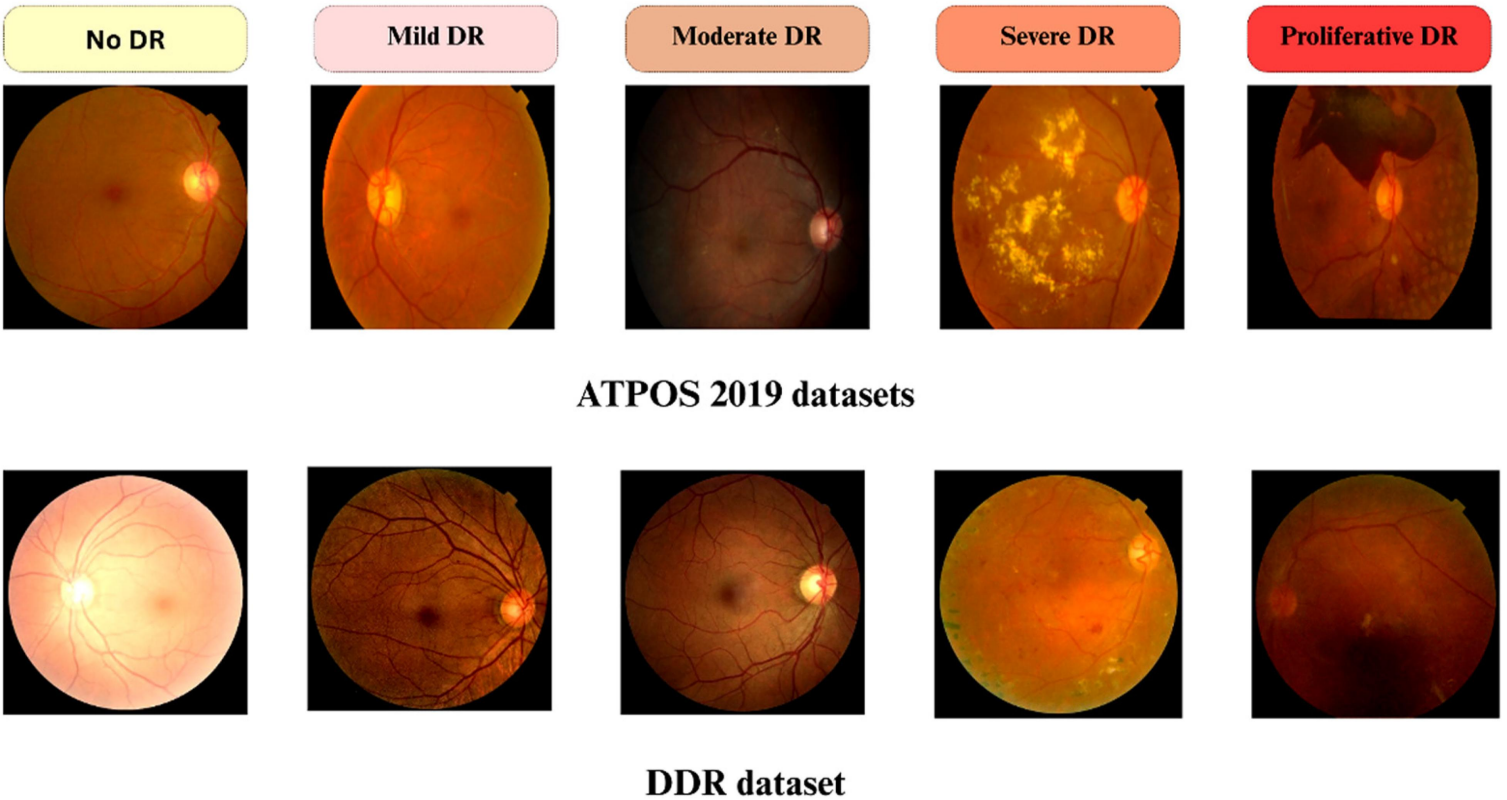
Sample images from APTOS 2019 and DDR datasets.

The image samples of each class have been presented in Figure 2. The images in the dataset have been divided into 5 classes- class_0, class_1, class_2, class_3, class_4 each corresponding to No DR, Mild Non-Proliferative DR (Mild NPDR), Moderate Non-Proliferative DR (Moderate NPDR), Severe Non-Proliferative DR (Severe NPDR) and Proliferative DR (PDR). The upper row consists of image from APTOS 2019 dataset and the below row consists of images from DDR dataset.

It should be noted that the datasets used in this study do not provide explicit patient-level identifiers. Therefore, data splitting is performed at the image level under the assumption of independence between samples. While stratified splitting is applied to preserve class distribution, patient-level separation could not be enforced.

### 4.2 Image preprocessing

Preprocessing helps make the images consistent, balance the classes, and allow the model to learn effectively. Table 2 shows the data augmentation parameters utilized for training the images. As described in the dataset section, the images are first grouped into folders according to their severity levels using the labels provided in the respective dataset CSV files. Based on this structure, two experimental setups are prepared. In the multi-class classification, the original five severity categories (No DR, Mild, Moderate, Severe, and Proliferative DR) are kept to enable detailed grading of disease progression, while in the binary classification, all diseased categories (Mild, Moderate, Severe, and Proliferative DR) are merged into a single class labeled as DR, with No DR kept as the second class. To balance the classes in the binary dataset, the prevalent class (No DR) is undersampled by randomly removing excess samples until it matches the size of the minority class. This ensures that both categories contribute equally during model training and minimizes bias toward the dominant class. For the multi-class classification setup, oversampling is performed through data augmentation to balance the underrepresented severity levels. An augmentation pipeline is executed using the Keras ImageDataGenerator API with transformations specified in Table 2, thereby adding variation and improving minority class representation. In addition, all images are resized to a fixed resolution of 224 × 224 pixels using the Pillow (PIL) library and converted to RGB format to maintain uniform channel consistency.

**Table 2:** Data augmentation parameters and their effects on training images for multiclass classification.

Parameter	Value	Description
zoom_range	0.1	Randomly zooms images by up to 10% during training.
rotation_range	15	Randomly rotates images within a 15-degree range.
horizontal flip	TRUE	Randomly flips images horizontally.
width_shift_ range	0.1	Shifts images horizontally by up to 10% of the width.
height_shift_ range	0.1	Shifts images vertically by up to 10% of the height.

Following preprocessing, the dataset is divided into training (80%), validation (10%), and testing (10%) sets using stratified sampling to maintain consistent class distribution across all subsets. To address class imbalance, different strategies are applied for binary and multiclass settings. In the binary classification setup, the majority class (No DR) is undersampled to match the minority class size, ensuring balanced representation. For multiclass classification, augmentation-based oversampling is applied to underrepresented classes using transformations such as rotation (15°), zoom (0.1), horizontal flipping, and width and height shifts (0.1), as specified in Table 2. All images are resized and normalized prior to training to ensure uniformity. The augmentation process is applied only to the training set, while validation and test sets remain unchanged to ensure unbiased evaluation and prevent any overlap between data subsets.

To ensure a fair evaluation and avoid data leakage, the dataset is first divided into training, validation, and testing sets using stratified sampling. This division is performed prior to any augmentation or class balancing. All augmentation techniques and oversampling methods are applied only to the training set, while the validation and test sets remain unchanged. This prevents overlap between subsets and ensures unbiased performance evaluation.

### 4.3 Proposed network

#### 4.3.1 Encoder

**Figure 3:**
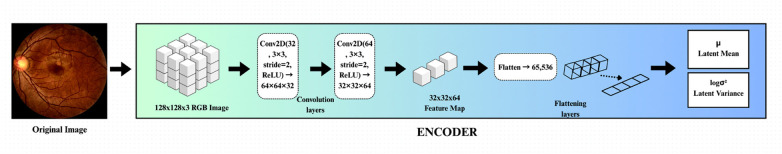
Proposed encoder network.

As per Figure 3, the proposed encoder design includes the following steps. First, a fundus image of size 128 × 128 pixels with 3 color channels is fed to the encoder. The encoder consists of convolutional filters that help in identifying and extracting patterns from the fundus images. Low-level features like edges and corners are captured by the first convolutional layer. The second convolutional layer is designed to extract the textures and shapes present in the image. The convolutional layers work on the approach of reducing the image size while retaining the important features that are essential for identification and classification. The feature-based reduced images of size 64 × 64 × 3 are then subjected to flattening, which ensures that a 2D matrix of features is efficiently converted into a long vector. This 1D image map is later used to develop a reconstructed image, which can be fed to a Logistic Regression classifier that can then work on the classification of the level of DR from the image.

The Variational Autoencoder (VAE) produces two outputs that are instrumental in aiding the classifier, which are the mean, *μ*, and the log of variance, log *σ*^2^. The mean is an indicator of the center of the distribution, and the variance measures how spread out the distribution is. Features such as lesions, microaneurysms, and hemorrhages can be represented through the mean. Variance highlights the uncertainty and variability present in the feature map. Here, z is the compressed representation, μ is the mean, *σ* is the variance, and *ε* is the random noise drawn from the standard normal distribution with mean 0 and variance 1. z is utilized by the decoder to capture the essential retinal features. z aids the Logistic Regression classifier in effective classification.

The sampling process involves the formation of a compressed representation of the image, which is represented by the formula as given in Equation 1 below.

$$z=\mu +\exp\;(0.5\cdot \log\;\sigma^{2}),\;\;\varepsilon ∼N(0,1)$$ (1)

Here in Equation 1, z is the compressed representation, μ is the mean, *σ* is the variance, and *ε* is the random noise drawn from the standard normal distribution with mean 0 and variance 1. z is utilized by the decoder to capture the essential retinal features. z aids the Logistic Regression classifier in effective classification.

#### 4.3.2 Decoder

**Figure 4:**
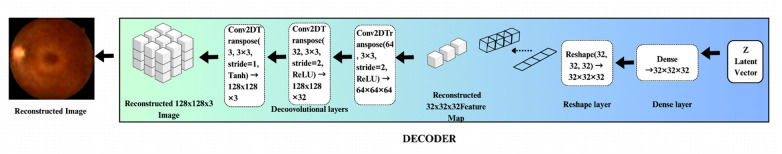
Proposed decoder network.

The decoder is designed to take z, the latent variable, as the input and then provide a reconstructed image that can be used by the Logistic Regression classifier to identify the various levels of DR. It is broadly divided into three parts: Input (z), Dense Layer Expansion, and Deconvolution. As presented in Figure 4, the decoder takes in the latent vector z as input, which undergoes dense layer expansion and reshaping, and then the reconstructed image map is passed through transpose convolutional layers to form the reconstructed images. The output from the encoder is a 1D vector feature map containing details from the image, such as haemorrhages, lesions, and microaneurysms. This output from the encoder acts as an input for the decoder, which then acts on the 1D factors, takes in the essential features, and reconstructs an image from the 1D vector by utilizing its deconvolutional (or transpose convolutional) layers. The reconstructed image containing the necessary spatial features can then be passed on to the Logistic Regression classifier. The 1D latent vector, z, is expanded by the dense layer. This dense layer essentially transforms the 1D latent vector into an expanded feature vector. The mentioned step is essential in ensuring the retention of spatial features and also because a direct application of deconvolutional layers on the 1D latent vector is not possible without using a dense layer. Transpose convolutional layers are applied to increase the spatial dimensions of the expanded feature vector step by step. It aims to provide a reconstructed image with major features like exudates and retinal textures in their original positions in the image but with an emphasized effect. This result is achieved as it learns where lesions, haemorrhages, and vessels should be placed in the image on the basis of the patterns, features, and details captured in the 1D latent vector while smoothing out the noise. Since the retinal images contain blood vessel curves and lesions with irregular shapes and textures, simple linear operations cannot be applied to capture and learn the intricate and detailed structures. A Rectified Linear Unit (ReLU) is used to introduce non-linearity into the network. ReLU provides an effective approach for the decoder to learn complex features that cannot be reduced to linear patterns or straight-line features. It also improves efficiency by making the network more effective and highlighting details crucial to classification.

f(x)=max(0,x) (2)

In function mentioned in Equation 2, the output retains its value if it is positive, and the output becomes 0 if the value fed into the function is negative. Conventionally used functions such as sigmoid or tanh result in problems that cause gradients to become very small during backpropagation. ReLU tackles this issue by not saturating for positive values, ensuring faster training and a more stable approach. ReLU is applied to assist the decoder in retaining information about shapes, textures, and intensity that are crucial in the formation of reconstructed images that resemble real fundus images, which are not oversimplified or flat, thus improving the training and testing accuracy of the model. ReLU is applied to each convolutional and deconvolutional layer for this purpose. The normalization of the final output images in the range [−1, 1] is done by the tanh activation function. This ensures that the output is on the same scale as the input image, making reconstruction easier. The goal of the decoder is to produce a reconstructed output image that is exactly the same size as the input fundus image. This enables direct comparison and analysis with the original image during training.

Unlike conventional VAE-based DR classification methods that directly utilize latent embeddings with deep neural classifiers, the proposed framework introduces a reconstruction-guided classification strategy. In this approach, reconstructed images are used as feature-refined representations for classification instead of relying solely on latent vectors. This enables the model to emphasize clinically relevant retinal features while suppressing noise and redundant variations. Furthermore, the combination of disentangled latent representations with a simple Logistic Regression classifier provides a balance between accuracy, interpretability, and computational efficiency, distinguishing it from existing approaches that depend on complex deep classifiers.

To ensure reproducibility, the encoder employs two convolutional layers featuring filter sizes of [X, Y] and a kernel size of [k × k], each followed by ReLU activation and a downsampling operation. The latent space dimension is fixed at 64, where distinct dense layers compute the mean and log-variance parameters. A corresponding decoder structure utilizes dense expansion followed by transpose convolutional layers with matching filter sizes and kernel configurations to reconstruct the image. ReLU activation is applied in all layers except the final one, which employs a tanh function for output normalization. These architectural decisions were selected to balance effective feature extraction against computational demands, aligning with the lightweight design goals of the proposed model.

#### 4.3.3 Model training

The training of the Variational Autoencoder (VAE) depends on a combination of losses that are essential to ensure accurate classification is achieved. The losses are broadly divided into four categories: Reconstruction Loss, KL Divergence Loss, Perceptual Loss, and FFT Loss.

##### 4.3.3.1 Reconstruction loss or main loss

The reconstruction loss accounts for the difference between the original and the reconstructed image. It ensures that the reconstructed image is highly similar to the original, with all the major disease-relevant features intact in z that could prove useful to the classifier and also highlights the disease severity in the image, making it easier to classify into various grades of DR. It attempts to measure the pixel-wise difference between the original image and the reconstructed output. It is given by the mean squared loss formula as specified in Equation 3:

$$L_{recon}=\frac{1}{N}\overset{N}{\underset{i=1}{\sum }}\;\|x_{i}-{\hat{x}}_{i}{\|}^{2}$$ (3)

In Equation 3, $x_{i}$ is the original pixel, ${\hat{x}}_{i}$ is the reconstructed pixel and N is the number of pixels.

##### 4.3.3.2 KL divergence loss

The KL Divergence Loss as shown in Equation 4 is applied to ensure that the encoder's latent vector, z, follows a Gaussian distribution. It is used to prevent overfitting of the model and enables sampling, which helps in generating new fundus images. It works by comparing the difference between the encoder output and the standard normal distribution N(0,1) and is given by:

$$L_{KL}=-\frac{1}{2}\overset{d}{\underset{j=1}{\sum }}\;(1+\log\;(\sigma_{j}^{2})-\mu_{j}^{2}-\sigma_{j}^{2})$$ (4)

In Equation 4, $\mu_{j}$ is the mean of latent variable j and $\sigma_{j}^{2}$ is variance of the latent variable j and d is the number of latent dimensions.

##### 4.3.3.3 Perceptual loss

The perceptual loss is required because MSE, or the Reconstruction Loss, only works by comparing exact values and may ignore intricate structures like textures. It works by comparing features extracted by a pretrained network such as VGG and maintains disease-related features and patterns. The mechanism of Perceptual Loss is given by extracting features from an intermediate CNN layer for both original and reconstructed images. Equation 5 expresses the perceptual loss as:

$$L_{perc}=\underset{l}{\sum }\;\|\phi_{l}(x)-\phi_{l}(\hat{x}){\|}^{2}$$ (5)

In Equation 5, $\phi_{l}(\cdot )$ is the feature map at layer l of the pretrained network.

##### 4.3.3.4 FFT loss

Similar to perceptual loss, the FFT Loss is a key factor in medical imaging applications. Lesions, which are crucial for identifying DR, often have sharp edges that might get blurred due to pixel-level loss. FFT Loss operates in the frequency domain by ensuring high-frequency details with sharp edges are preserved by converting the images into the frequency domain using the Fast Fourier Transform (FFT) and comparing the original and reconstructed images. It is described in Equation 6:

$$L_{fft}=\|F(x)-F(\hat{x})\|$$ (6)

In Equation 6, F(⋅) Is the Fourier transform of the image.

##### 4.3.3.5 Total loss

The total loss which is obtained from the combination of all four losses is given in Equation 7 below:

$$L_{total}=L_{recon}+L_{perc}+L_{fft}+\beta L_{KL}$$ (7)

In Equation 7, β is the disentanglement factor and its value is set to 4.0.

The Equation 7 depicts a combination of reconstruction, KL divergence, perceptual, and FFT losses were chosen to ensure that both pixel-level fidelity and clinically relevant structural details are preserved. The *β* value was empirically selected as 4 to balance reconstruction quality and latent space disentanglement, as confirmed through preliminary experiments and ablation analysis. This configuration was found to provide optimal performance across both datasets.

#### 4.3.4 Classification

The output of the Beta VAE is a reconstructed image, which is then flattened into a 1D vector. The Equation 8 shows how this 1D vector retains pixel-level information extracted from the fundus image, which then acts as the input to the Logistic Regression classifier.


$$s=w^{T}z+b$$ (8)

The probability function given in Equation 8 is of the Logistic Regression classifier is computed using the linear score, which is given as, where w is the weight vector, b is the bias, and z is the input feature or the latent vector. This score then acts as the input to the sigmoid function (for binary classification), which calculates its probability between 0 and 1. The sigmoid function is shown in Equation 9.

$$P(y=1\;|\;z)=\sigma (s)=\frac{1}{1+e^{-s}}$$ (9)

If the probability is greater than 0.5, then the sample is classified as Class 1 (DR Present); otherwise, it is marked as Class 0. In multilevel classification of DR, we classify the images into five clinical levels. These levels are Class 0, Class 1, Class 2, Class 3, and Class 4, which correspond to No DR, Mild Non-Proliferative DR (Mild NPDR), Moderate NPDR, Severe NPDR, and Proliferative DR, respectively. Equation 10 shows the probability function for multiclass DR can be given as:

$$P(y=k\;|\;z)=\frac{e^{w_{k}^{t}z+b_{k}}}{\sum_{j=0}^{K}\;e^{w_{j}^{t}z+b_{j}}}$$ (10)

In Equation 10, k corresponds to the levels of DR, ranging from 0 to 4 and z is the latent vector and $w_{k}$ is the weight for the class ‘k’ and $b_{k}$ is the bias for the class ‘k’. The class with the maximum probability is selected.

## 5 Experimentation

The model training and testing were done using Google Colab Pro with an NVIDIA Tesla T4 GPU. The dataset for binary classification on the APTOS 2019 dataset consists of 1,825 samples of class_0 (No DR) and 1,867 samples of class_1 (DR) of colored fundus images, resized to 128 × 128 pixels and normalized to the [0, 1] range. In the case of the DDR dataset, there are 1,825 class_0 images and 1,867 class_1 images. An 80/20 split is used to form the training and testing sets and is used for hold-out validation, while additional evaluation is performed using 5-fold stratified cross-validation to tackle class imbalance. The model was trained for 80 epochs to ensure convergence.

The training procedure follows a consistent pipeline across all experiments. The dataset is first split into training, validation, and testing sets using stratified sampling to preserve class distribution. Data augmentation is applied exclusively to the training set to prevent information leakage. The *β*-VAE is first trained to convergence, after which reconstructed images are generated and used to train the Logistic Regression classifier. This two-stage training ensures that feature learning and classification are decoupled, improving stability and interpretability.

The model training setup is quite similar for the multiclass classification, with images being divided into class_0, class_1, class_2, class_3, and class_4, which correspond to No DR, Mild NPDR, Moderate NPDR, Severe NPDR, and PDR, respectively. The APTOS 2019 dataset consists of 1,817 samples in each class, whereas the DDR dataset consists of 2,000 samples in class_0 and 1,849 samples in the rest of the classes. The model was trained under similar conditions as the binary classifier model but with 80 epochs.

Table 3 summarizes the hyperparameters utilized for training the binary classifier on the APTOS 2019 and DDR datasets. For both datasets, input images were resized to a fixed resolution of 128 × 128 pixels. The batch size was taken as 32, and the number of epochs per fold was taken as 80 for both datasets. The Adam optimizer was used, and the value of the disentanglement factor was taken as 4. Similarly, in Table 4, the hyperparameters employed in training the multiclass classifier for the APTOS 2019 and DDR datasets are described, with the batch size being 8 and 2,000 images per class in both datasets. The number of epochs per fold was taken as 30, and the value of the beta factor was taken as 4, similar to the binary classifier arrangement. The experimentation was conducted with beta being 4 as well as 1 (standard VAE), with the best results being obtained when beta was taken as 4. The value of 4 maintains a balance between disentanglement and better reconstruction quality.

**Table 3:** Hyperparameters taken for binary classifier.

Hyperparameters	Values for ATPOS 2019 (binary-class)	Values for DDR (binary-class)
Image size	(128, 128)	(128, 128)
Latent dimension (VAE)	64	64
Batch size	32	32
Training instances	1,825 + 1,867 = 3,692	2,000 + 2,000 = 4,000
Training split	80% Training	80% Training
Testing split	10%	10%
Validation split	10% (from training split)	10% (from training split)
Optimizer	Adam	Adam
Epochs per fold	80	80
Loss function	VAE Loss (Reconstruction + β KL)	VAE Loss (Reconstruction + β KL)
β (KL weight)	4	4
Cross-validation	5-fold Train/Test split with validation split	5-fold Train/Test split with validation split
Classifier	Logistic Regression	Logistic Regression

**Table 4:** Hyperparameters taken for multiclass classifier.

Hyper parameters	Values for ATPOS 2019 (multi-class)	Values for DDR (multi-class)
Image size	(128, 128, 3)	(128, 128, 3)
Latent dimension (VAE)	64	64
Batch size	8	8
Training instances	2,000 (each class)	2,000 (each class)
Number of Classes	5 classes (balanced)	5 classes (balanced, 0–4)
Training split	80% Training	80% Training
Testing split	10%	10%
Validation split	10% (from training split)	10% (from training split)
Optimizer	Adam (learning rate = 1e-4)	Adam (learning rate = 1e-4)
Epochs per fold	80	80
Loss function	VAE Loss (Reconstruction + β KL)	VAE Loss (Reconstruction + β KL)
β (KL weight)	4	4
Cross-validation	5-fold Train/Test split with validation split	5-fold Train/Test split with validation split
Classifier	Logistic Regression (multinomial)	Logistic Regression (multinomial)

## 6 Results and discussions

This section details the proposed model's performance, comparing its classification accuracy and computational efficiency against baseline and state-of-the-art models. The evaluation is conducted across two distinct datasets: APTOS 2019 and DDR. The training, validation and testing curves show a consistent and stable trend of convergence through epochs, with all three curves taking a similar path and only a small gap between them. This strong correlation suggests that the model is learning generalizable patterns and not memorizing the training data, thus indicating low levels of overfitting. In particular, validation and test accuracies are in accordance with training accuracy during the entire training process without major fluctuations or divergence, further confirming the stable learning behavior. In addition, the robustness of the model is confirmed by the results of the 5-fold cross-validation, where the performance is consistent across the data splits. The model also shows similar high performance on two independent datasets (APTOS 2019 and DDR), on both binary and multiclass classification settings. The consistency of the results across different evaluation protocols and datasets provide strong evidence that the model can generalize and less likely overfitting or dataset-specific bias. While the proposed framework enhances interpretability through feature-focused reconstruction, it does not explicitly localize lesions. Future work will integrate explainability methods such as Grad-CAM or SHAP to provide clinically actionable visual explanations.

### 6.1 Performance analysis

This section discusses the results of the proposed model on binary and multi-class DR classification using the APTOS 2019 and DDR datasets. The performance of the model is evaluated using parameters such as accuracy, precision, recall, F1-score, specificity, sensitivity, and the Receiver Operating Characteristic (ROC) curve and AUC values. This section is broadly divided into parts that elaborate on the results of the model in binary classification as well as multiclass classification of DR. The performance of the proposed model has been compared with a similar model without the disentanglement factor (beta) and also with a simple Logistic Regression classifier to note the improvement in results. The efficiency of the proposed model has also been examined by comparing it with the results obtained by different models on the same dataset, giving a clear idea of the classification accuracy of the mod.

**Figure 5:**
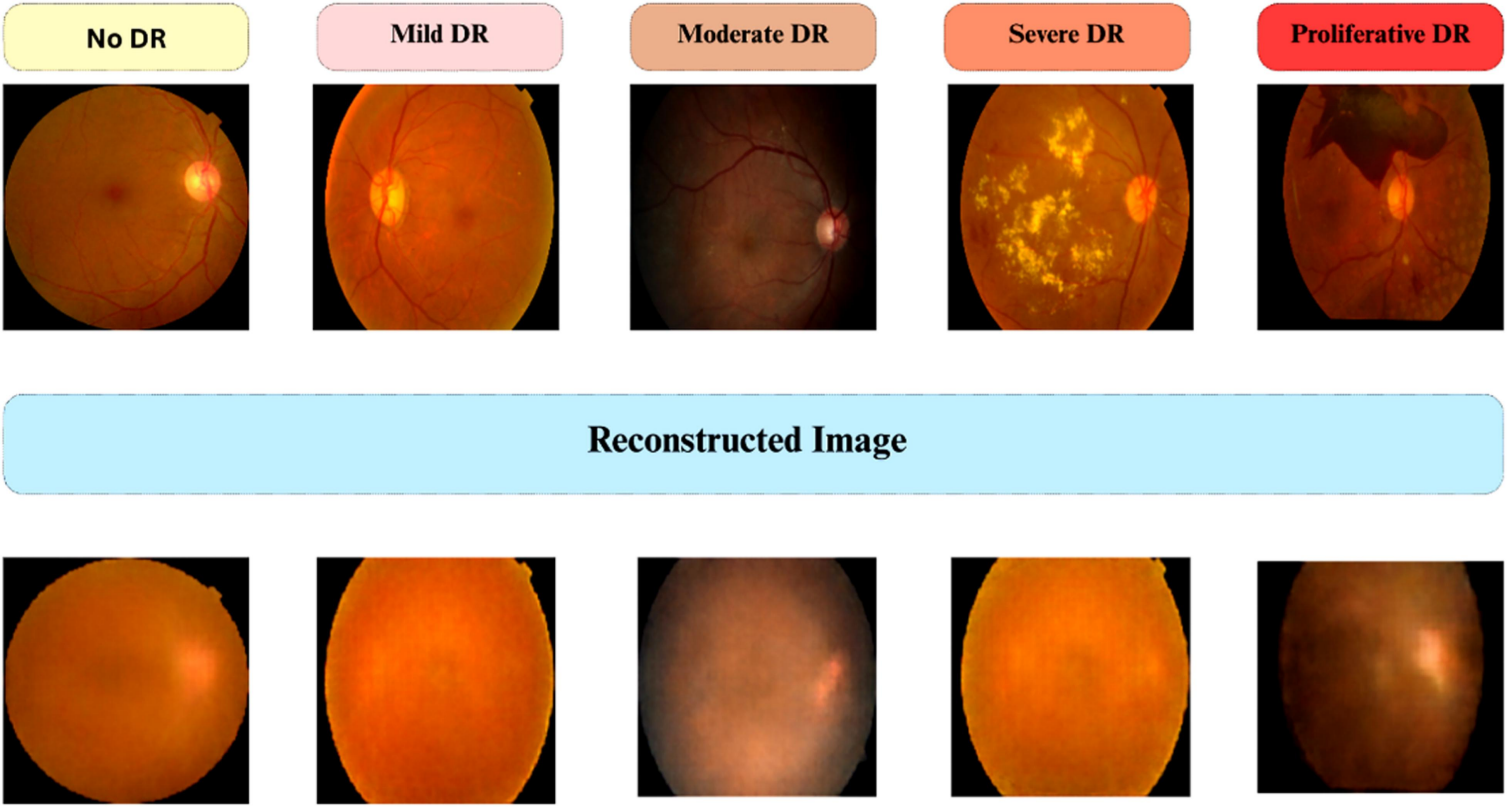
Sample images and their reconstructed output.

In Figure 5, the upper row displays the original image samples whereas the below row presents the reconstructed images obtained from the VAE.

The reconstructed images generated by the *β*-VAE provide a form of feature-level interpretability by emphasizing clinically relevant structures such as microaneurysms, hemorrhages, and exudates while suppressing background noise. This allows the classifier to operate on simplified representations that retain diagnostically meaningful information. However, it is important to note that this interpretability is implicit and does not correspond to explicit lesion localization or attribution.

#### 6.1.1 Binary classification

**Figure 6:**
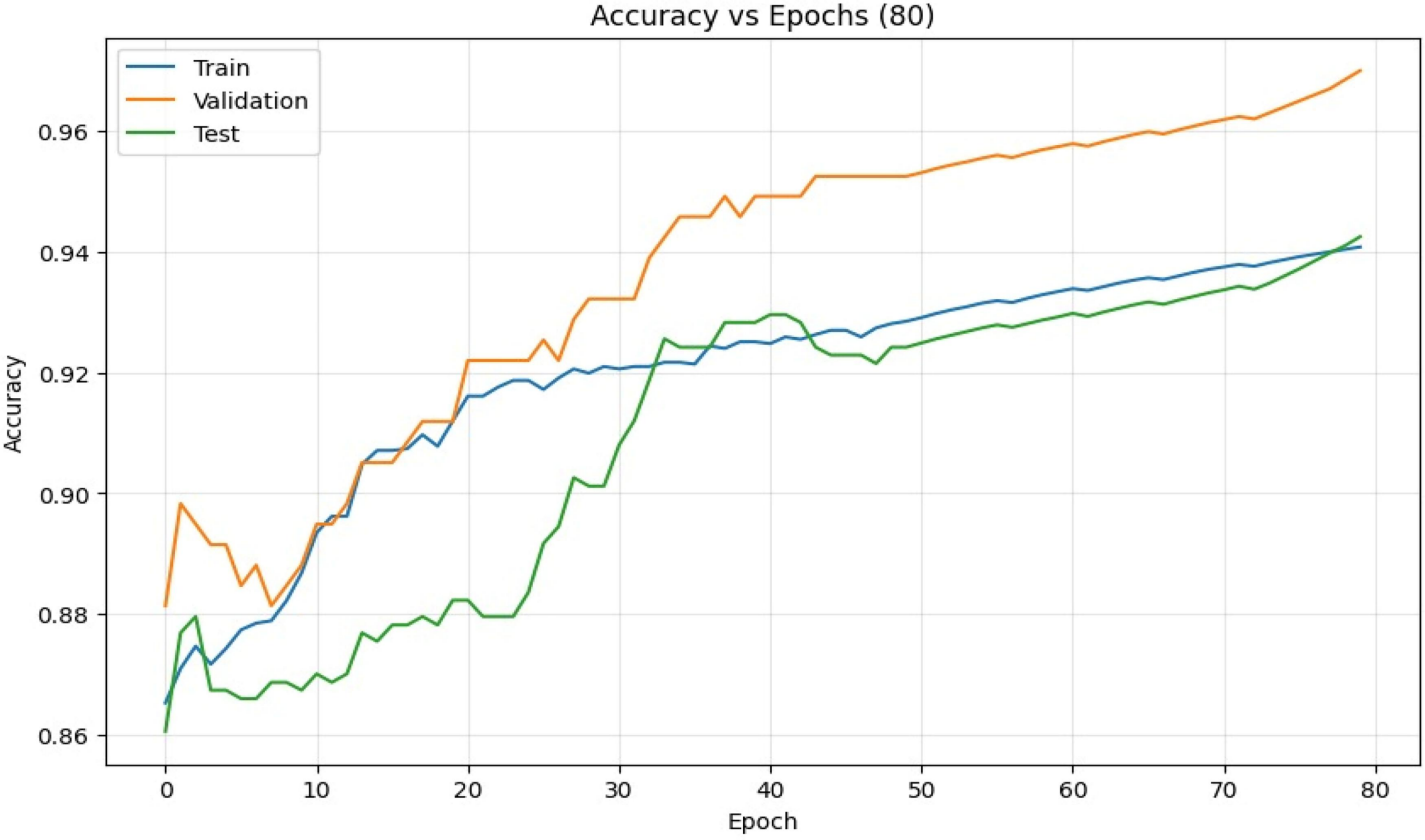
Training vs. testing vs. validation curve for APTOS 2019 binary classification.

**Figure 7:**
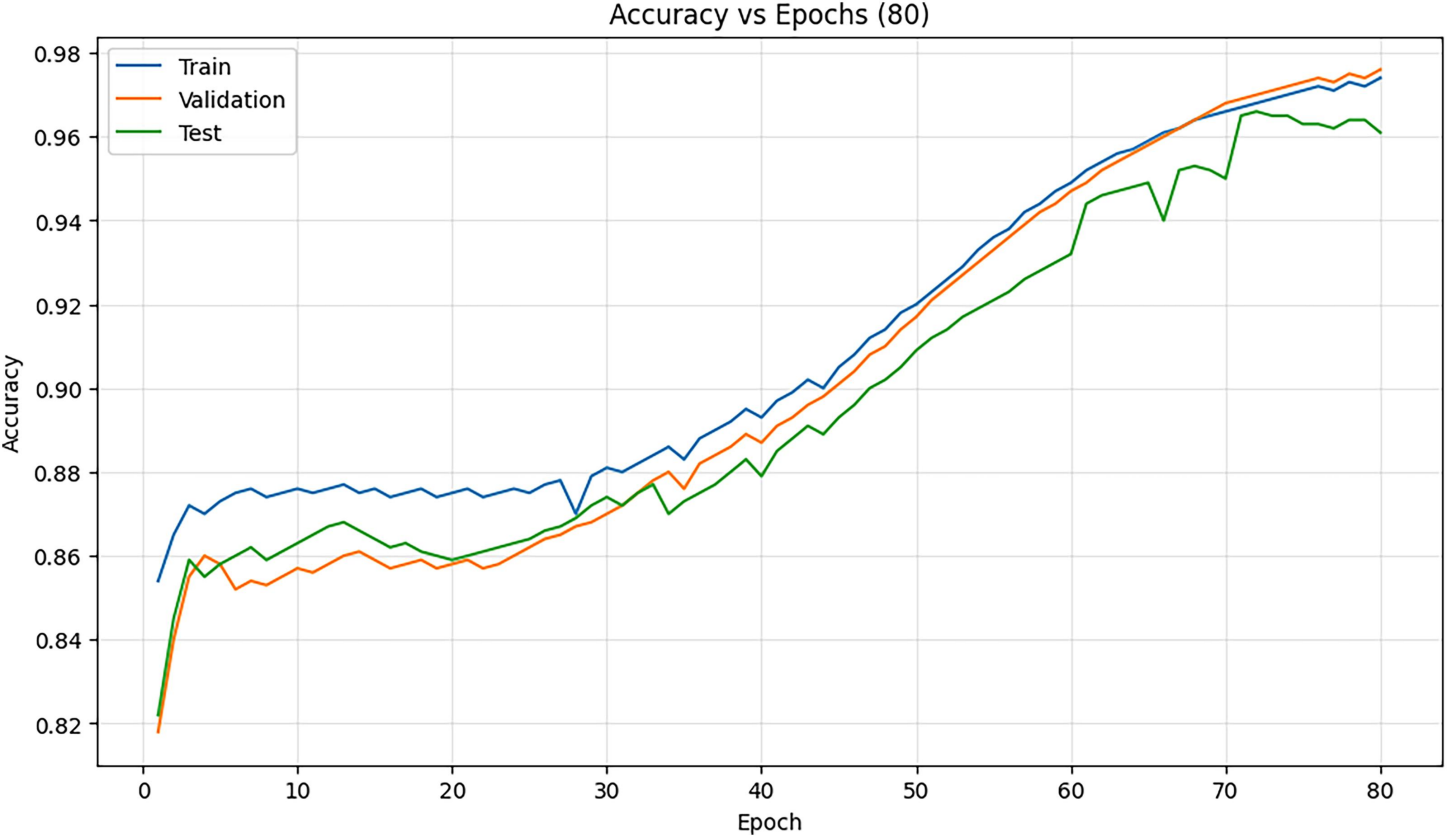
Training vs. testing vs. validation curve for DDR Dataset.

The binary model is first evaluated on binary classification of identifying the images and identifying it in 2 classes, class 0 and class 1 which correspond to the categories of No DR and DR respectively. The model achieved an accuracy of 98.64% with a precision of 98.65% and recall of 98.64% on APTOS 2019 Dataset. The model also obtained similar results on the DDR datasets with an accuracy of 97.83%, precision of 97.86% and a recall of 97.83% which indicates that the proposed model is highly capable of identifying a high proportion of disease cases while also maintaining a suitable false positive rate. Training, validation, and test accuracy variations over 80 epochs are shown in Figures 6, 7, where all three curves show a steady and consistent improvement. The model exhibits stable learning with little overfitting as it progressively converges over 80 epochs, with training accuracy approaching nearly 97% and validation and test accuracies stabilizing around 94%–95%.

**Figure 8:**
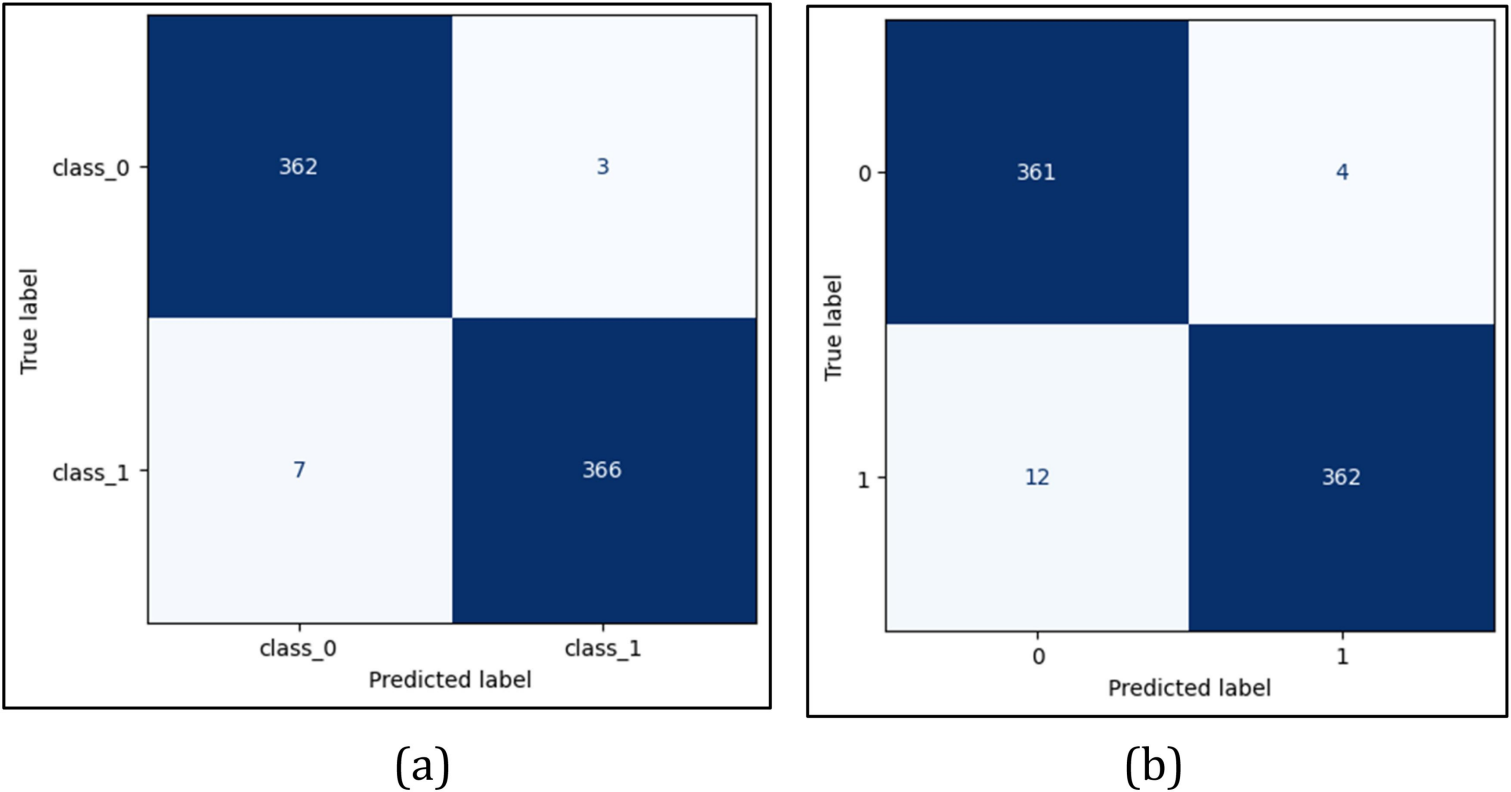
**(a)** confusion matrices of binary classification on APTOS 2019; **(b)** confusion matrices of binary classification on DDR.

**Figure 9:**
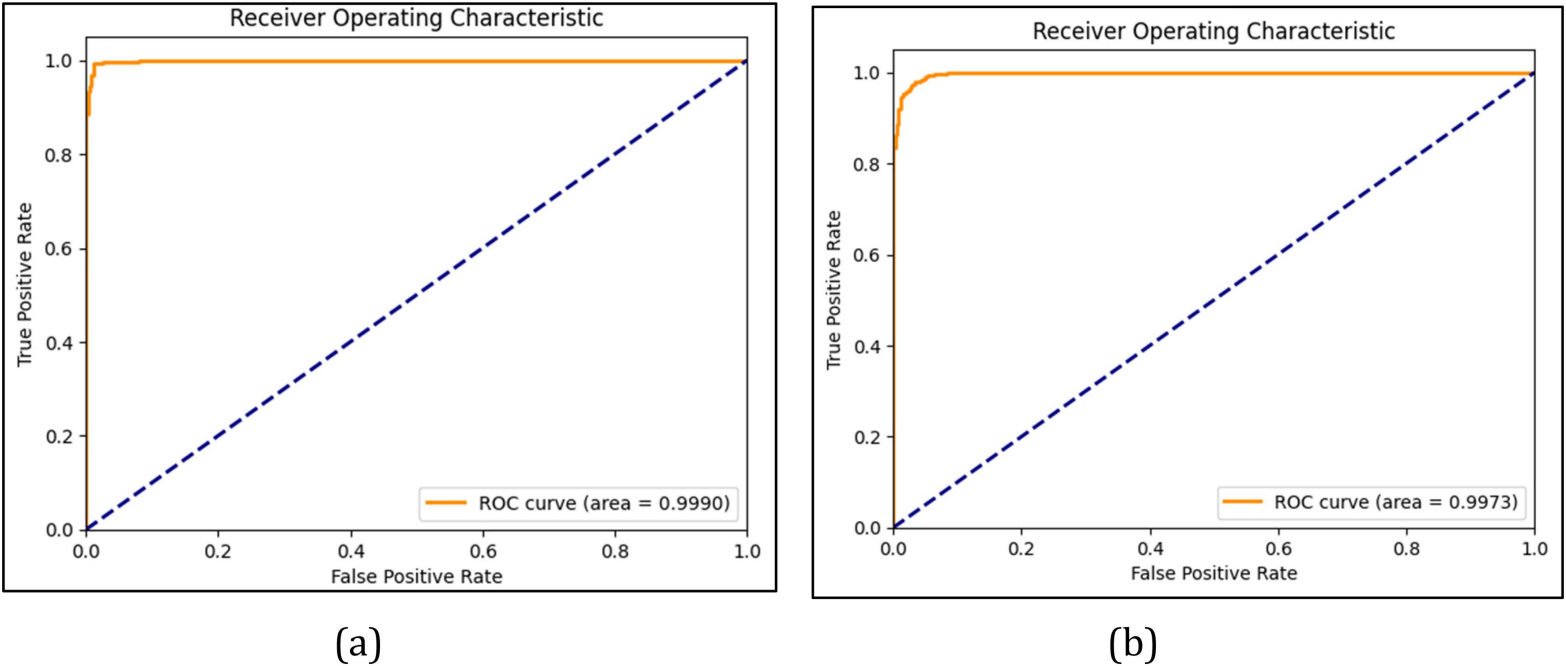
**(a)** ROC curves of binary classification on APTOS 2019; **(b)** ROC curves of binary classification on DDR.

A thorough examination of the confusion matrices in Figures 8a,b suggests that the model has been successful in reducing false negatives, which are crucial in clinical settings where missing a DR case may have unforeseen repercussions. While specificity deals with the reliable identification of No DR images, the high sensitivity attained guarantees that DR cases are appropriately classified. The model's consistency in handling DR is demonstrated by the comparable metrics produced after being trained on two different datasets. The findings imply that the model performs well by retaining essential feature-based information while eliminating extraneous details and random noise, concentrating primarily on colors, textures, shapes, and other important details. Additional validation using the ROC curves in Figures 9a,b reveals an AUC value of 0.99 on both datasets, demonstrating the robust discriminative power of the suggested framework. Also, the model's consistent performance and ability to handle the slight class imbalance in the datasets are confirmed by the F1-score, which offers a balanced measure of precision and recall. All of these findings show that the suggested method produces robust classification performance in both binary and multiclass settings, as well as dependable convergence and strong generalization.

#### 6.1.2 Multiclass classification

**Figure 10:**
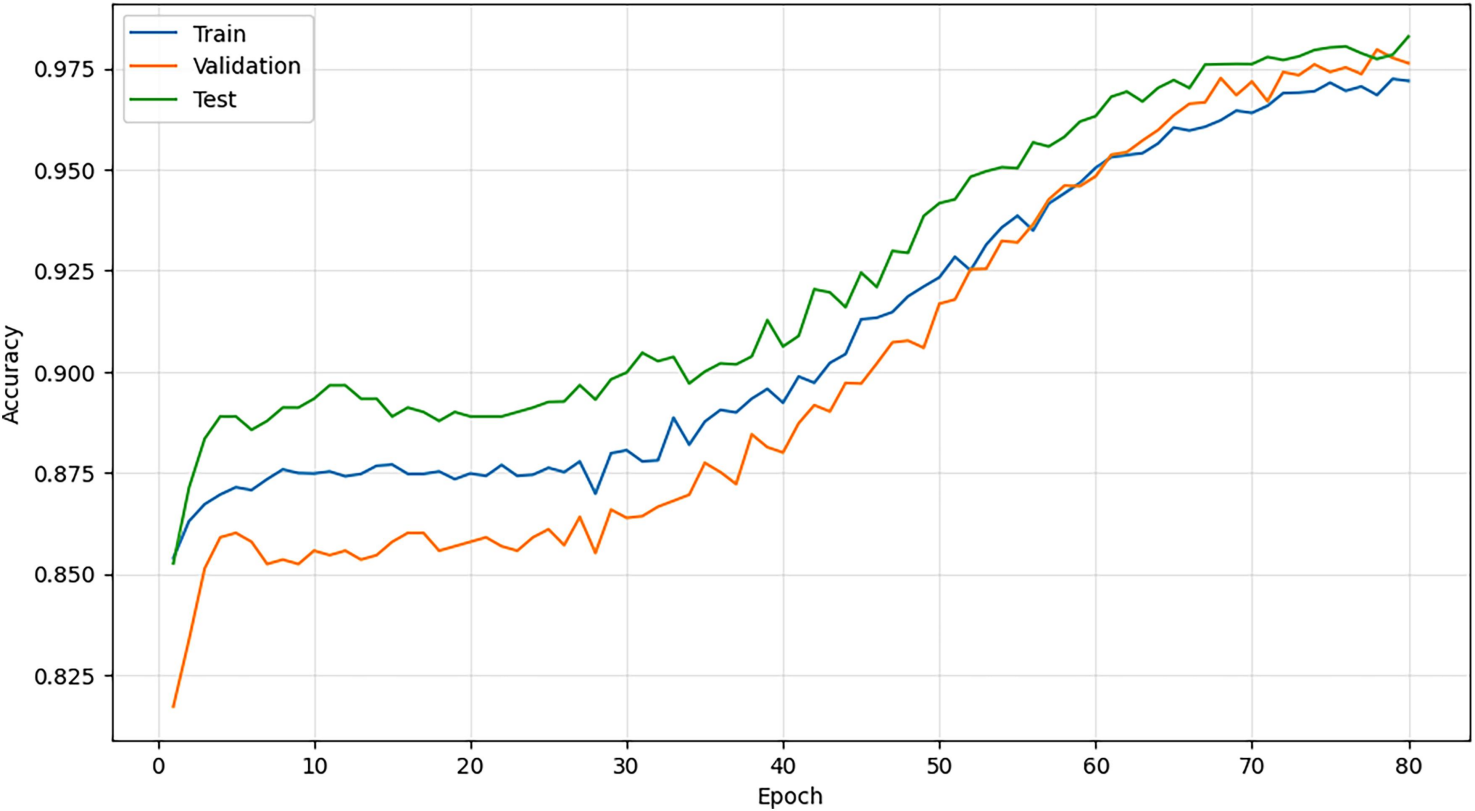
Training vs. testing vs. validation curve for APTOS 2019 multiclass classification.

**Figure 11:**
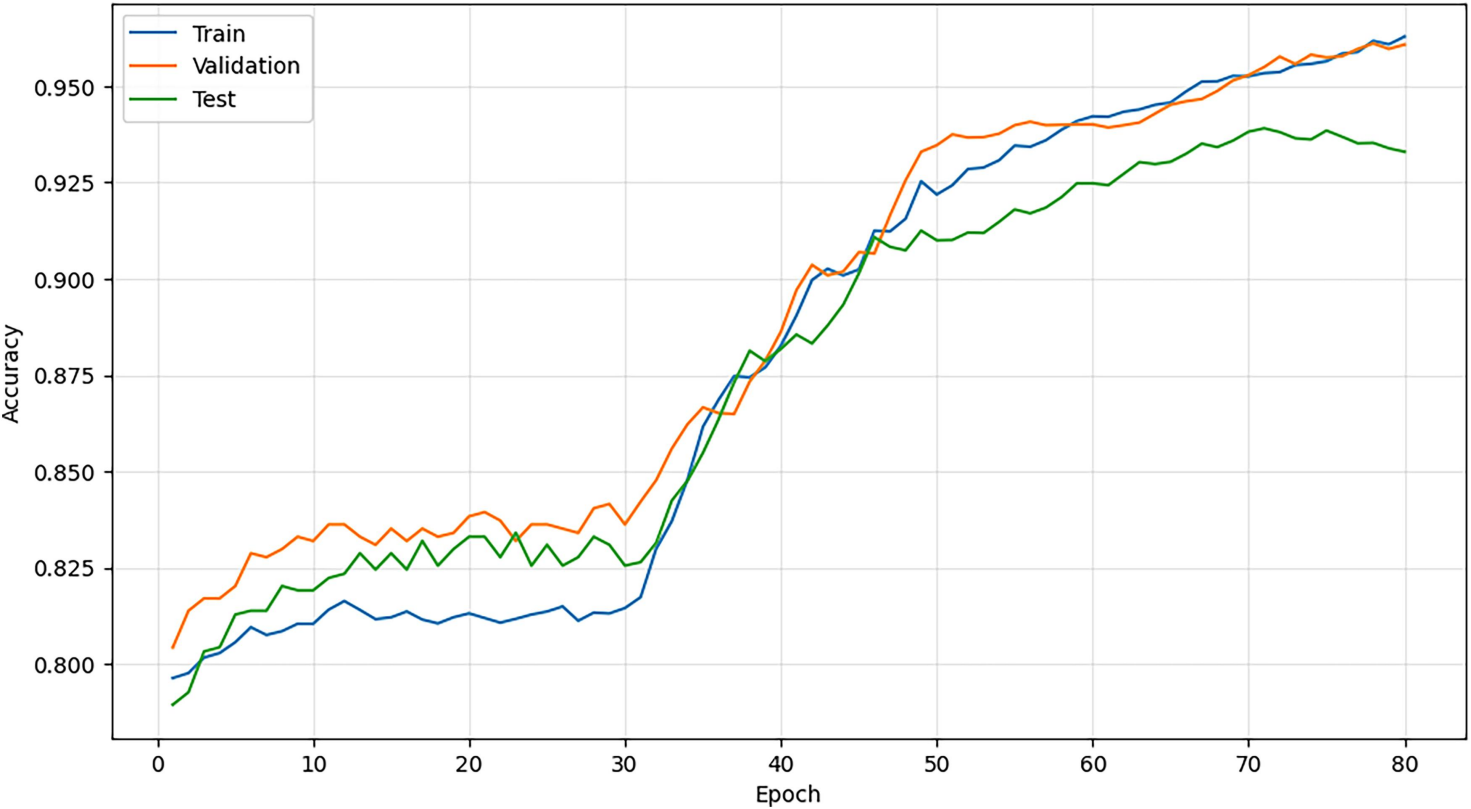
Training vs. testing vs. validation curve for DDR multiclass classification.

For the multiclass classification, the proposed model was modified accordingly to expand its classification domain from 2 classes to 5 with categories being No DR, Mild, Moderate, Severe, and Proliferative DR. The model achieved an overall accuracy of 97.80% with a precision of 97.84% and the recall being 97.80% when evaluated on the APTOS 2019 Dataset. Similar performance was observed for DDR dataset with an accuracy of 97.23%, precision of 97.42% and the recall being 97.26%. The F1 score across both datasets showed the ability of the model to identify each class in a balanced manner despite the challenges of class imbalance and the similarities between adjacent DR classification levels. Training, validation, and test accuracy trends over 80 epochs for each experimental setting are shown in Figures 10, 11. The accuracy curves show a steady upward trend in both situations, suggesting steady and advanced learning behaviour. The validation and test curves follow a similar trajectory, stabilizing just below the training curve, while the training accuracy steadily increases and converges near 97%–98%. Strong generalization across unseen data and little overfitting are suggested by the comparatively narrow gap between the three curves. A more gradual and smoother increase is seen in the first graph, which shows consistent optimization over the course of training. When the model captures the discriminative latent features, the curves in the second graph show effective convergence after a sharper rise around the mid-epochs. The suggested Beta VAE-based framework's resilience and consistency in acquiring significant retinal representations for precise DR classification are further supported by the aligned behaviour of training, validation, and test accuracies in both figures.

**Figure 12:**
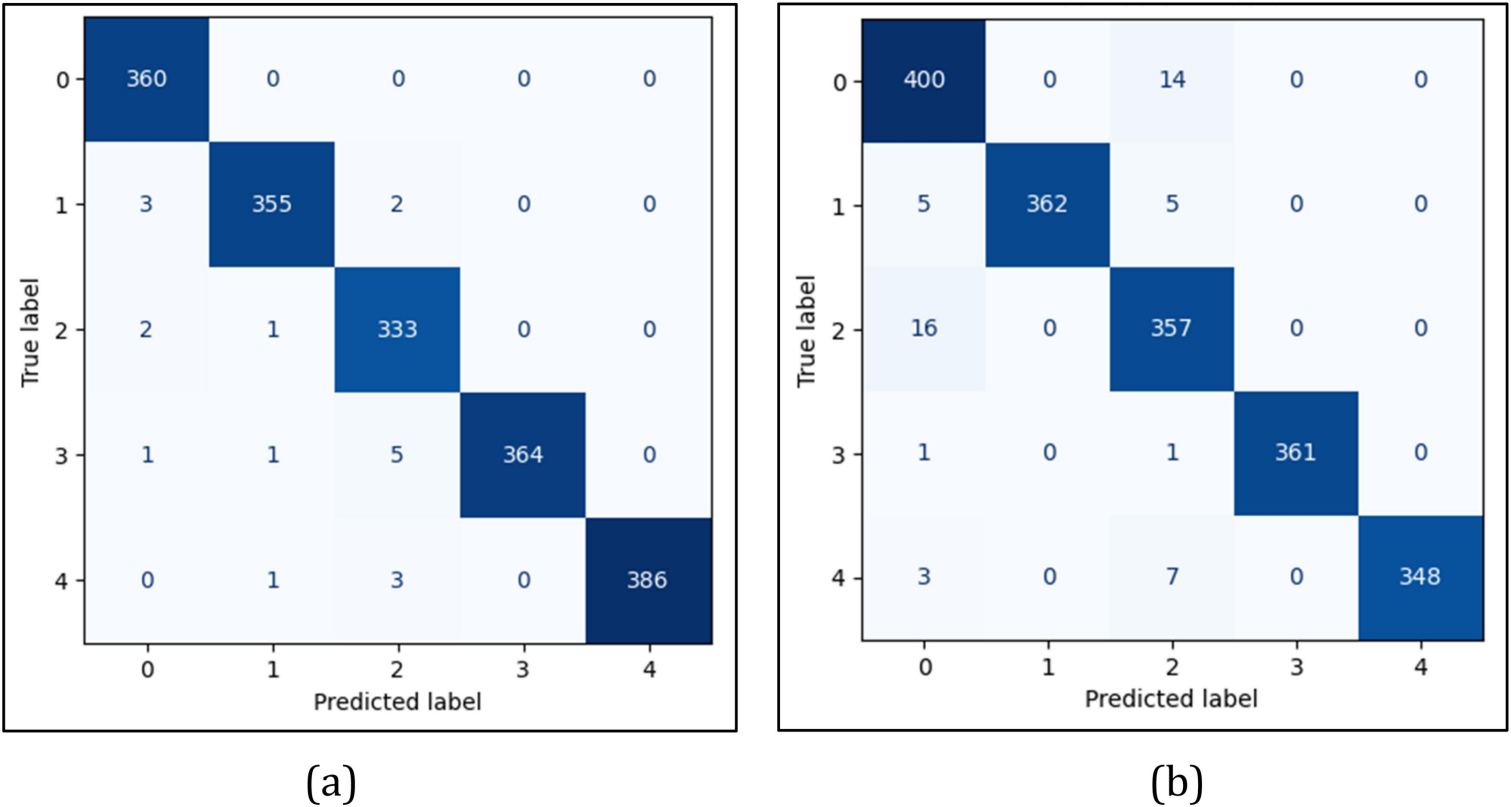
**(a)** confusion matrices of multiclass classification on APTOS 2019; **(b)** confusion matrices of multiclass classification on DDR.

**Figure 13:**
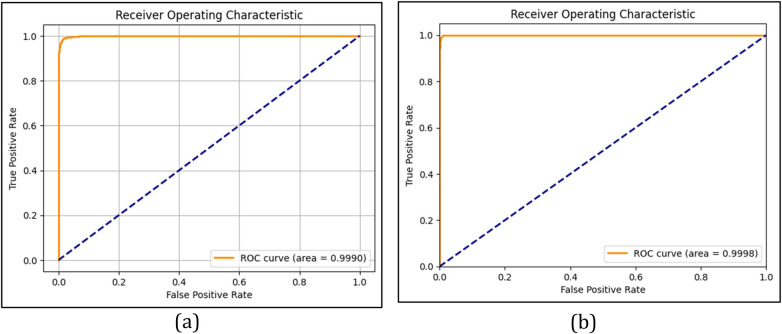
**(a)** ROC curves of multiclass classification on APTOS 2019; **(b)** ROC curves of multiclass classification on DDR.

An inspection of the confusion matrices from Figures 12a,b revealed that some of the classification errors occurred between neighbouring stages such as mild and moderate or severe and proliferative dr, which is a consequence of the minute differences present in the features of the fundus images belonging to these classes. The latent representation vectors, which capture the fine details crucial for classification, enable the model to achieve deeper and more accurate separation of classes compared to raw pixel-based approaches. The positive performance of the model on both datasets in multiclass dr classification as well as in binary classification, along with high values of the evaluation metrics, indicates the ability of the model to reliably predict true positives, thereby reducing the risk of undiagnosed dr or false positives. The roc curves and high auc values (0.98–0.99) indicate the enhanced ability of the model to distinguish between classes. The ROC curves presented in Figures 13a,b yield an AUC value of 0.99 on both datasets, reflecting the strong discriminative capability of the model and indicating effective and well-optimized training.

### 6.2 Ablation study

The effect of the disentanglement factor, or the beta factor, was examined by testing it on the APTOS 2019 dataset and the DDR dataset. Tables 5, 6 shows the binary classification results for the APTOS 2019 and DDR dataset respectively. Tables 7, 8 shows multiclass classification results for APTOS 2019 and DDR dataset respectively. As per the results shown in these tables, the baseline Logistic Regression classifier without the VAE only achieved an accuracy of 84% and 82% in binary and multiclass classification. The performance improved slightly by pairing the Logistic Regression classifier with a standard Variational Autoencoder, achieving accuracies of 94% and 92% in binary and multiclass classification. The VAE and Logistic Regression (LR) model, powered by the disentanglement factor, achieved greater results as mentioned before. Also, the model worked well in comparison to a custom classifier combined with a VAE, which achieved accuracies of 91% and 88% in binary and multiclass classification, whereas the custom classifier with Beta VAE resulted in accuracies of 92% and 89%. In the case of the DDR dataset, LR achieved 76.68% and 75.56% accuracy in binary and multiclass classification, which increased to 96% and 94% with the integration of a standard VAE. The Beta VAE and LR again resulted in a better-performing model, with the results highlighted before. In comparison, the custom classifier with a standard VAE achieved 84% and 80% accuracy, and with Beta VAE achieved 85% and 82%, which are lower than Beta VAE coupled with LR. The disentanglement factor, or the beta factor, in the Beta VAE significantly improves the quality and distinguishing capability of the latent vector produced by the Beta VAE. The scaling of the KL Divergence term in the VAE loss function improves two major deciding factors by enhancing reconstruction accuracy, ensuring the reconstructed images are similar to the original images, and enabling the latent variables to be independent and well-organized. The disentanglement is necessary to consider as DR severity is computed by localized and minute patterns such as Microaneurysms, Haemorrhages, Hard and Soft exudates and texture details. Also, the disentanglement factor enables the VAE to retain these features while tackling the challenges of brightness caused by poor imaging equipment, noise in the images and irrelevant structures in the DR.

**Table 5:** Comparison of results from binary classification on different models on APTOS 2019 dataset.

Dataset & classification	APTOS 2019 binary classification											
Type of validation	Hold out cross validation (%)						5-fold cross validation (%)					
Model	Acc	Prec	Recall	F1	Spec	Sens	Acc	Prec	Recall	F1	Spec	Sens
LR	84.01	85.92	81.77	83.79	86.30	81.77	84.53	87.03	80.72	84.07	88.44	80.72
VAE + LR	94.31	94.38	94.31	94.31	96.16	92.49	94.56	94.59	94.55	94.56	95.62	93.52
**Beta VAE + LR**	**98.64**	**98.65**	**98.64**	**98.65**	**99.18**	**98.12**	**97.94**	**97.95**	**97.94**	**97.94**	**98.63**	**97.27**
VAE + Custom classifier	91.88	91.88	91.88	91.88	92.05	91.71	91.20	91.20	91.21	91.20	92.48	91.93
Beta Vae + Custom classifier	92.56	92.57	92.56	92.56	93.15	91.98	92.42	92.43	92.42	92.41	92.99	91.86

Here the bold values indicate the highest value recorded for the corresponding evaluation metrics while doing the experiment.

**Table 6:** Comparison of results from binary classification on different models on DDR dataset.

Type of validation	Hold out cross validation (%)						5-fold cross validation (%)					
Model	Acc	Prec	Recall	F1	Spec	Sens	Acc	Prec	Recall	F1	Spec	Sens
LR	78.62	78.78	78.62	78.60	81.92	75.40	76.68	76.78	76.68	76.66	78.30	75.09
VAE + LR	95.75	96.35	94.73	95.53	96.69	94.73	96.09	96.70	95.36	96.02	96.78	95.36
**Beta VAE** **+** **LR**	**97**.**83**	**97**.**86**	**97**.**83**	**97**.**83**	**98**.**90**	**97**.**83**	**97**.**00**	**97**.**00**	**97**.**32**	**97**.**01**	**96**.**66**	**97**.**32**
VAE + Custom classifier	84.42	84.48	84.42	84.41	86.30	82.57	84.05	84.11	84.05	84.04	85.81	82.32
Beta Vae + Custom classifier	87.26	87.34	87.26	87.26	89.32	85.25	85.72	85.8	85.72	85.72	87.52	83.97

Here the bold values indicate the highest value recorded for the corresponding evaluation metrics while doing the experiment.

**Table 7:** Comparison of results from multiclass classification on different models on APTOS 2019 dataset.

Type of validation	Hold out cross validation (%)						5-fold cross validation (%)					
Model	Acc	Prec	Recall	F1	Spec	Sens	Acc	Prec	Recall	F1	Spec	Sens
LR	81.51	82.52	81.51	81.84	95.38	81.51	82.83	83.59	82.83	83.08	95.71	82.83
VAE + LR	93.40	93.60	93.40	93.43	93.85	93.40	92.80	93.13	92.80	92.86	96.20	92.80
**Beta VAE** **+** **LR**	**97**.**80**	**97**.**84**	**97**.**80**	**97**.**81**	**99**.**45**	**97**.**80**	**98**.**28**	**98**.**32**	**98**.**28**	**98**.**29**	**99**.**57**	**98**.**28**
VAE + Custom classifier	88.60	89.15	88.61	88.71	97.15	88.61	88.18	88.20	88.17	88.34	96.37	88.18
Beta Vae + Custom classifier	89.43	90.01	89.43	89.52	97.35	89.43	89.11	89.92	89.11	89.25	97.28	89.11

Here the bold values indicate the highest value recorded for the corresponding evaluation metrics while doing the experiment.

**Table 8:** Comparison of results from multiclass classification on different models on DDR dataset.

Type of validation	Hold out cross validation (%)						5-fold cross validation (%)					
Model	Acc	Prec	Recall	F1	Spec	Sens	Acc	Prec	Recall	F1	Spec	Sens
LR	75.69	76.26	75.69	75.79	93.89	75.85	75.56	75.97	75.56	75.48	75.56	75.69
VAE + LR	94.57	94.97	94.55	94.70	98.63	94.55	94.35	94.92	94.32	94.51	98.57	94.32
**Beta VAE** **+** **LR**	**97**.**23**	**97**.**42**	**97**.**26**	**97**.**32**	**99**.**30**	**97**.**26**	**97**.**49**	**97**.**66**	**97**.**48**	**97**.**54**	**99**.**36**	**97**.**48**
VAE + Custom classifier	81.21	84.00	81.71	81.99	95.26	81.17	80.53	83.17	80.58	81.46	94.35	80.58
Beta Vae + Custom classifier	82.75	85.22	82.99	83.75	95.67	82.91	82.80	84.39	82.66	83.73	95.42	82.85

Here the bold values indicate the highest value recorded for the corresponding evaluation metrics while doing the experiment.

To further analyze the contribution of individual loss components, additional experiments were conducted using different loss configurations for multiclass classification, as shown in Table 9. When only reconstruction loss (MSE) is used, the model achieves an accuracy of 82.93% on APTOS 2019 and 77.91% on the DDR dataset, indicating that pixel-level similarity alone is insufficient for effective feature learning. When the disentanglement factor is reduced to *β* = 1 (standard VAE), the performance improves but remains lower than the proposed *β* = 4 configuration. The best performance is achieved with *β* = 4, demonstrating the importance of disentangled latent representations.

**Table 9:** Effect of different loss configurations on classification accuracy for APTOS 2019 and DDR datasets.

APTOS 2019 (multiclass)			DDR (multiclass)		
Model	Loss function	Accuracy	Model	Loss function	Accuracy
Beta VAE + LR	MSE (Reconstruction Loss Only)	0.8293	Beta VAE + LR	MSE (Reconstruction Loss Only)	0.7791
Beta VAE + LR	Beta=1	0.9280	Beta VAE + LR	Beta=1	0.9435
Beta VAE + LR	Beta=4	**0**.**9828**	Beta VAE + LR	Beta=4	**0**.**9749**

Here the bold values indicate the highest value recorded for the corresponding evaluation metrics while doing the experiment.

Additionally, when individual loss components such as perceptual loss, FFT loss, or KL divergence are applied in isolation, the model exhibits overfitting and unstable convergence. This indicates that each component alone is insufficient, and their combination is necessary to ensure balanced learning of global structure, high-frequency details, and latent space regularization. These results validate the effectiveness of the combined loss formulation used in the proposed model.

### 6.3 Statistical analysis

The one-way ANOVA results presented in Tables 10, 11 demonstrate that the differences in performance among the evaluated models are statistically significant. For the APTOS 2019 dataset, the computed F-value of 11.21 with a corresponding *p*-value of 2.27 × 10⁻⁶ is substantially below the significance threshold of 0.05. Similarly, for the binary classification setup, an F-value of 52.46 with a *p*-value of 1.68 × 10⁻⁸ is obtained, which is also highly significant. In ANOVA, a high F-value indicates that the variation between model performances is much greater than the variation within each model, suggesting that the models do not perform equally. The extremely low *p*-values in both cases confirm that these differences are not due to random chance. Therefore, the null hypothesis, which assumes that all models have similar performance, is rejected. These findings provide strong statistical evidence that the proposed Beta-VAE with Logistic Regression model performs significantly differently and better than the baseline approaches. The ANOVA results are consistent with the observed improvements in the evaluation metrics and validate the effectiveness of the proposed framework. Overall, this confirms that the performance gains achieved by the proposed model are both reliable and statistically meaningful.

**Table 10:** One-way ANOVA results for model comparison on APTOS 2019 dataset.

Source	SS	df	MS	F	Prob > F
Between Groups	526.687	4	131.672	5.979	0.0,00,455
Within (Error)	1211.201	55	22.022	—	—
Total	1737.888	59	—	—	—

**Table 11:** One-way ANOVA results for model comparison on DDR dataset.

Source	SS	df	MS	F	Prob > F
Between Groups	889.829	4	222.457	3.556	0.011911
Within (Error)	3440.98	55	62.563	—	—
Total	4330.809	59	—	—	—

### 6.4 SOTA analysis

Various models have utilized the APTOS 2019 dataset, ranging from conventional CNNs to advanced deep learning frameworks. In Table 12, where various models have been compared, it is seen that VGG16 paired with an XGBoost classifier obtained an accuracy of 79.5%, whereas an accuracy of 87.43% has been reported by a DenseNet-201 classifier. New and emerging models such as decision tree-based ensemble learning and EfficientNet-B7 achieved up to 94.20% and 84% accuracy, with the best result being 94.64% accuracy recorded by the SHAP explanations. In addition to these models, recent studies (2023–2025) have explored more advanced architectures such as transformer-based models, hybrid CNN frameworks, and explainable AI-integrated systems for diabetic retinopathy classification, demonstrating improved feature representation and generalization capabilities (26, 27). These approaches are considered strong baselines due to their ability to capture global contextual features and handle large-scale image variability effectively. In contrast to the models in Table 12, the proposed model achieved an accuracy of 97% and 98% in binary and multiclass classification, respectively, giving better performance than the aforementioned models. It highlights the ability of the model to capture and analyze both major and fine-grained retinal features, enabling efficient classification among the various classes of DR.

**Table 12:** Comparison of results from binary and multiclass classification and different models on APTOS 2019 dataset.

Ref. No	Dataset used: APTOS 2019	Accuracy	
	Model	Binary	Multi-class
	**Proposed Model**	**98.64%**	**97.80%**
(8)	VGG16	92.50%	66.70%
(9)	Inception V3	N/A	81.61%
(10)	DenseNet-169	N/A	90%
(11)	DenseNet201	N/A	87.43%
(12)	Xception CNN model	98.36%	84.36%
(13)	EfficientNet-B7	N/A	84.00%
(14)	decision tree-based ensemble learning technique	N/A	94.20%
(17)	Source-Free Transfer Learning (SFTL) model	N/A	91.20%
(18)	SHapley Additive exPlanations (SHAP).	N/A	94.64%

Here the bold values indicate the accuracy achieved by the proposed methodology which is highest among the others.

Similar to the APTOS dataset, recent works have also evaluated modern architectures on the DDR dataset, including lightweight CNNs, hybrid deep learning models, and memory-efficient frameworks such as MEDCNet, which have shown strong performance in large-scale medical imaging tasks (16). The DDR dataset has also been thoroughly analyzed and tested by various models, frameworks, and approaches, as shown in Table 13. Previously, models such as MobileNet and MobileNetV2 reported accuracies of 71% and 79% (binary), while more detailed algorithms such as DEC and CNN512 achieved 88%–89% accuracy. The best accuracy recorded is 96.01% using the DVE algorithm. In comparison to the few results mentioned above, the proposed model has achieved an accuracy of 97% in both binary and multiclass classification. This consistency indicates that the 1D latent vector produced by the Beta VAE is successful in its ability to generalize the variations and differences in fundus images in the DDR dataset. These results confirm the robustness of the proposed model and indicate its high usability in real-world applications.

**Table 13:** Comparison of results from binary and multiclass classification and different models on DDR dataset.

Ref. No	Dataset used: DDR	Accuracy	
	Model	Binary	Multi-class
	**Proposed Model**	**97.83%**	**97**.**23%**
(7)	CNN512	N/A	88.60%
(7)	CNN512 +YOLOv3	N/A	89%
(15)	FCM (algorithm)	N/A	83.80%
(15)	K-Means (algorithm)	N/A	87.40%
(15)	DEC	N/A	88.80%
(16)	MEDCNet	N/A	95.92%
(25)	Inception-v3	N/A	82.84%

Here the bold values indicate the accuracy achieved by the proposed methodology which is highest among the others.

It is important to note that the comparison with existing models is based on reported results from the literature. While all methods are evaluated on the same benchmark datasets, differences in preprocessing, data splitting, and training configurations across studies may influence performance. Therefore, the comparison is intended to provide a general performance reference rather than a strictly controlled experimental benchmark.

The use of reconstructed feature-enhanced images enables clearer representation of disease-relevant structures such as microaneurysms and hemorrhages, which are critical for diagnosis. Additionally, the interpretability offered by the Logistic Regression classifier allows clinicians to better understand the model's decision-making process, increasing trust and usability in real-world settings.

## 7 Conclusion

The results of this study demonstrate the effectiveness of the feature-focused learning mechanism with a simple yet effective classifier, which can prove to be a useful alternative to conventional deep learning approaches for classification. The Variational Autoencoder with the disentanglement factor, or beta factor, improves the accuracy of the model and also enables the model to retain fine details while tackling major challenges caused by external factors such as brightness or poor imaging equipment. The similar results obtained by the model on both the APTOS 2019 and DDR datasets indicate the ability of the model to adapt and effectively classify the images. The lightweight nature of the model enables faster training with reduced costs, which is very useful in real-world applications.

From a clinical perspective, the proposed framework offers a lightweight and efficient solution for automated diabetic retinopathy screening, making it particularly suitable for deployment in resource-constrained settings such as primary care centres and rural clinics. The use of reconstructed feature-focused images reduces computational complexity while retaining diagnostically relevant information, enabling faster inference on low-cost hardware.

In terms of deployment, the model can be integrated into computer-aided diagnosis systems to assist ophthalmologists by providing preliminary screening and severity grading. However, real-world implementation would require additional validation on diverse clinical datasets, robustness to variations in imaging conditions, and integration with explainability tools to support clinical decision-making. These aspects represent important directions for future work toward clinical translation.

While the proposed framework provides feature-level interpretability through reconstruction and disentangled latent representations, it does not explicitly perform lesion localization or provide attribution maps. Future work will focus on integrating explainability techniques such as Grad-CAM, SHAP, or attention-based visualization methods to highlight specific pathological regions and enhance clinical trust and usability. Additionally, validation of reconstructed features with clinical experts will further strengthen the interpretability of the model.

## Data Availability

Publicly available datasets were analyzed in this study. This data can be found here: Publicly available datasets were analyzed in this study. These datasets can be found at: https://www.kaggle.com/competitions/aptos2019-blindness-detectionhttps://github.com/nkicsl/DDR-dataset.
